# Relation of Insulin Resistance to Brain Glucose Metabolism in Fasting and Hyperinsulinemic States: A Systematic Review and Meta-analysis

**DOI:** 10.1210/clinem/dgae570

**Published:** 2024-08-24

**Authors:** Nicole J Jensen, Ane J Porse, Helena Z Wodschow, Helene Speyer, Jesper Krogh, Lisbeth Marner, Michael Gejl, Albert Gjedde, Jørgen Rungby

**Affiliations:** Department of Endocrinology, Copenhagen University Hospital Bispebjerg, 2400 Copenhagen, Denmark; Steno Diabetes Neuro Unit, Translational Type 2 Diabetes Research, Clinical Translational Research, Steno Diabetes Center Copenhagen, 2730 Herlev, Denmark; Department of Endocrinology, Copenhagen University Hospital Bispebjerg, 2400 Copenhagen, Denmark; Steno Diabetes Neuro Unit, Translational Type 2 Diabetes Research, Clinical Translational Research, Steno Diabetes Center Copenhagen, 2730 Herlev, Denmark; Steno Diabetes Neuro Unit, Translational Type 2 Diabetes Research, Clinical Translational Research, Steno Diabetes Center Copenhagen, 2730 Herlev, Denmark; Copenhagen Research Centre for Mental Health—CORE, Mental Health Centre Copenhagen, Faculty of Health Science, University of Copenhagen, 2100 Copenhagen, Denmark; Steno Diabetes Neuro Unit, Translational Type 2 Diabetes Research, Clinical Translational Research, Steno Diabetes Center Copenhagen, 2730 Herlev, Denmark; Clinic for Pituitary Disorders, Department of Medicine, Zealand University Hospital, 4600 Køge, Denmark; Department of Clinical Physiology and Nuclear Medicine, Copenhagen University Hospital Bispebjerg, 2400 Copenhagen, Denmark; Department of Clinical Medicine, University of Copenhagen, 2200 Copenhagen, Denmark; Department of Biomedicine, Aarhus University, 8000 Aarhus, Denmark; Department of Neuroscience, Faculty of Health and Medical Sciences, University of Copenhagen, 2200 Copenhagen, Denmark; Translational Neuropsychiatry Unit, Department of Clinical Medicine, Aarhus University, 8200 Aarhus, Denmark; Department of Neurology and Neurosurgery, McGill University, Montreal, Quebec H3A 0G4, Canada; Steno Diabetes Neuro Unit, Translational Type 2 Diabetes Research, Clinical Translational Research, Steno Diabetes Center Copenhagen, 2730 Herlev, Denmark; Department of Clinical Medicine, University of Copenhagen, 2200 Copenhagen, Denmark

**Keywords:** insulin resistance, type 2 diabetes, obesity, brain glucose metabolism, brain glucose uptake

## Abstract

**Context:**

Abnormal brain glucose metabolism may cause cognitive disease in type 2 diabetes, yet the relation between insulin resistance and brain glucose metabolism has not been systematically described.

**Objective:**

We evaluated the impact of metabolic condition (fasting vs insulin stimulation, eg, from hyperinsulinemic clamp) on the association between insulin resistance of different etiologies and brain glucose metabolism.

**Data Sources:**

PubMed, Embase, Cochrane Library, and Web of Science were systematically searched from inception until February 2022.

**Study Selection:**

Of 656 unique records, we deemed 31 eligible. Criteria were studies assessing brain glucose metabolism (uptake or metabolic rate) by ^18^F-2-fluoro-2-deoxy-D-glucose-positron emission tomography in individuals characterized by measures of or clinical proxies for insulin resistance (eg, type 2 diabetes and obesity).

**Data Extraction:**

Two independent investigators extracted data and assessed study quality.

**Data Synthesis:**

We applied random-effects models to pool Hedge's g standardized mean differences. Insulin resistance was associated with decreased brain glucose metabolism during fasting [−0.47 SD, 95% confidence interval (CI): −0.73 to −0.22, *P* < .001, *I*^2^ = 71%] and increased metabolism during insulin stimulation (1.44 SD, 95% CI 0.79 to 2.09, *P* = .002, *I*^2^ = 43%). Contrary to type 2 diabetes and other insulin resistance-related conditions, obesity was not associated with brain hypometabolism in fasting states (0.29 SD, 95% CI −.81 to 1.39).

**Conclusion:**

Metabolic conditions modify associations between insulin resistance and brain glucose metabolism; ie, most individuals with insulin resistance display hypometabolism during fasting and hypermetabolism during insulin stimulation.

Type 2 diabetes and midlife obesity are associated with cognitive impairment affecting memory, processing speed, and executive functions (1, 2); both conditions increase the risk of Alzheimer's disease and other dementias (3-5). With the global increase in the prevalence of diabetes and obesity together with a prolonged lifespan, cognitive complications to metabolic disorders constitute a growing healthcare problem (6). The pathophysiology connecting metabolic and neurodegenerative diseases remains incompletely understood. However, diminished brain insulin sensitivity in obesity, diabetes, and dementia (7) has sparked interest in the impact of insulin resistance in brain and periphery on brain metabolism and function.

In Alzheimer's disease, cognitive decline and clinical symptoms are strongly correlated with reduced brain glucose uptake in cingulate, frontal, parietal, and temporal cortices (8, 9). ^18^F-2-fluoro-2-deoxy-D-glucose positron emission tomography ([^18^F]FDG PET) is the gold standard for measuring brain glucose uptake and utilization and is widely used for diagnosis of Alzheimer's disease. Importantly, brain glucose uptake measured by [^18^F]FDG PET reflects neuronal activity in heath and disease (10), which is also reflected in the close relationship between brain glucose uptake and flow signals from magnetic resonance imaging (11). Acquisition and analysis methods for [^18^F]FDG PET data differ substantially. Static (single-image) acquisition yields semiquantitative glucose uptake estimates using the standardized uptake value, which assesses cumulative FDG concentrations in regions of interest relative to the injected dose or a reference region (12). Conversely, dynamic (temporal series of images) acquisition provides quantitative uptake and phosphorylation estimates of FDG and potentially glucose itself when applying a correction factor (the “lumped” constant), through arterial blood sampling and analytical techniques like graphical methods (eg, the Gjedde-Patlak plot), spectral analyses, or compartment modeling (13, 14). Estimates from the different methods differ in nature but are tightly correlated with brain glucose uptake (13) and are often reported interchangeably. Here, the term brain glucose metabolism (BGM) covers both simple FDG uptake and quantitative estimates of glucose uptake and utilization.

The impact of insulin on BGM has long been considered negligible, as the predominant glucose transporters at the blood-brain barrier and in neuronal cells (GLUT1 and GLUT3) are insulin-independent (15). Early [^18^F]FDG PET studies report absent brain responses to hyperinsulinemia (16). However, the identification of insulin receptors and insulin-sensitive GLUT-4 transporters in the human brain (17, 18) has prompted a reevaluation of these early observations. Interestingly, insulin at fasting and below fasting levels seems to regulate brain glucose uptake (19), a response that is attenuated in persons with insulin resistance (20), indicating the presence of brain and concurrent peripheral insulin resistance.

Lately, several studies have addressed the association between insulin resistance and BGM in metabolic and neurodegenerative diseases (21). Some report Alzheimer's-like glucose hypometabolism with increasing peripheral insulin resistance in type 2 diabetes and prediabetes (22), while others find peripheral insulin resistance to be the strongest predictor for enhanced BGM during insulin stimulation from hyperinsulinemic clamps (23). Thus, insulin resistance seems to influence BGM in a manner that is modulated by the metabolic condition and/or level of insulin.

Given the diagnostic significance of [^18^F]FDG PET assessments in neurodegeneration and the increased risk of cognitive impairment in type 2 diabetes and obesity, the complex relationship between peripheral insulin resistance and BGM needs clarification. Differences in study populations, imaging methodologies, and examined metabolic conditions challenge the interpretation of existing literature. The objective of this systematic review and meta-analysis is (1) to describe the associations between insulin resistance of different etiology (eg, type 2 diabetes and obesity) and BGM in both fasting and insulin-stimulated states and (2) to test by meta-regression the hypothesis that insulin and glucose levels influence these associations.

## Materials and Methods

The protocol for this systematic review and meta-analysis was preregistered at PROSPERO (CRD42022309231; https://www.crd.york.ac.uk/prospero/display_record.php?ID=CRD42022309231).

### Data Sources and Search

The databases PubMed (MEDLINE), EMBASE, Cochrane Central Register of Controlled Trials (CENTRAL), and the Science Citation Index (Web of Science) were searched from inception until February 24, 2022. The search strategy was designed to capture studies on insulin resistance and related conditions and BGM and contained the concepts insulin resistance-related condition (eg, type 2 diabetes), [^18^F]FDG PET-imaging (eg, FDG), and brain (eg, cerebral). Search strings were manually translated between databases and used medical subject headings and text words or similar when possible [for search terms, see Supplementary Table S1 (24)]. Reference lists of included studies and relevant reviews were screened for additional studies.

### Study Selection

Two investigators (A.J.P. and N.J.J.) independently performed the literature screening first based on title and abstract followed by full-text screening using predefined criteria. Disagreements were solved by a third investigator (J.R.). Inclusion criteria were as follows: (1) individuals undergoing a brain scan allowing for subsequent (semi-)quantification of BGM; (2) individuals with a disease or syndrome characterized by insulin resistance (eg, type 2 diabetes, obesity, metabolic syndrome, or polycystic ovary syndrome) or individuals with an insulin resistance/sensitivity assessment (eg, homeostatic model assessment for insulin resistance or M-value); (3) brain scans were allowed to be conducted in both fasting and hyperinsulinemic states; (4) treatment with glucose-lowering drugs was allowed with no constraints on glycemic control, associated comorbidities, or related medication. Studies were excluded if (1) participants were <18 years, (2) participants were treated with systemic glucocorticoids, or (3) nonhuman species were examined. Reviews, case reports, letters, conference abstracts, and trial registrations were excluded, but no language restriction was applied. In instances where reports investigated identical or overlapping study populations, the report with the highest participant number or original studies with comprehensive study details as opposed to reports including pooled data were selected.

### Data Extraction

The following data were extracted independently by 2 investigators (A.J.P and N.J.J) using a structured and piloted spreadsheet: study characteristics [first author, year of publication, study design (meta-analysis included only cross-sectional data), sample size, and metabolic condition (fasting vs insulin stimulation)], participant characteristics [study population, sex, age, body mass index (BMI), fasting glucose level, insulin sensitivity, and cognitive status], PET protocol (quantification method, input function, brain region), and outcome assessment and potential mediators (brain FDG or glucose uptake/utilization value and insulin and glucose levels obtained during PET scans or close to scan time in fasting studies). The original paper was referred to when discrepancies in the extracted data occurred, and any subsequent disagreements were solved by a third investigator (J.R).

Outcome data from all reported brain regions were extracted; only 1 measure, prioritized as follows, was included in the primary analysis: whole brain, region within frontal lobe, temporal lobe, parietal lobe, occipital lobe, and subcortical regions. This decision was supported by similar trends among brain regions [Supplementary Fig. S1 (24)]. If more than 1 measure was reported within the same brain region, the means and corresponding SD were averaged. When outcome was reported for both hemispheres, left hemisphere data were extracted. Outcome data available only from figures (7 studies) were extracted by both investigators (A.J.P. and N.J.J.; mean interrater variability of 1.3%, range: 0-7%), using the online software WebPlotDigitizer (25), following Cochrane Handbook guidelines. The average of the 2 values was used for analysis. In 1 report, with discrepant values in text and figure, values were averaged. Reports providing only *P-*values for the association were excluded from the meta-analysis. As only few reports were intervention trials, only cross-sectional data at baseline was included in the meta-analysis.

Authors were contacted for unreported data or additional details when necessary.

### Quality Assessment

The methodological quality of included studies was assessed with the Joanna Briggs Institute Critical Appraisal Tool for Analytical Cross-Sectional Studies. This consists of 8 questions with response options “yes,” “no,” “unclear,” or “not applicable” and evaluates the study's consideration of potential bias in design, conduct, and analysis (26). Studies with <50% “yes” answers are deemed high risk, 50% to 69% moderate risk, and ≥70% low risk. Two independent investigators (A.J.P. and N.J.J.) conducted assessments, with disagreements resolved by a third investigator (J.R.).

### Data Synthesis and Analysis

Meta-analyses were performed in R Statistical Software (v4.3.0; R Core Team 2023) using the *meta* package (v6.5.0). Between-group effect sizes and corresponding SEs of BGM were calculated using Hedges’ G standardized mean differences (SMD). This enables comparisons across studies applying different methodologies for estimating BGM and corrects Cohen's d estimates for small study bias (27). Missing means and SDs were approximated from medians, interquartile ranges, and/or ranges using default methods in the *meta* package. Studies reporting Pearson or Spearman rank correlation coefficients (*r*) were transformed into Cohen's d formulas: $d=\frac{2r}{\sqrt{1-r^{2}}}$ and $SE_{d}=\sqrt{\frac{4Vr}{{\left(1-r^{2}\right)}^{3}}}$, where $V_{r}=\frac{{\left(1-r^{2}\right)}^{2}}{n-1}$ and further into Hedges’ G using the correction factor: $J=1-\frac{3}{4df-1}$ (28). If outcome was reported as the regression coefficient from a linear regression, it was transformed into Cohen's d by dividing the coefficient with its SD (29) after which Hedges’ G correction factor was applied. To ensure comparability between studies, the direction of the association was reversed in studies reporting an association for insulin sensitivity.

A random-effects model was used to pool effect sizes, as considerable between-study heterogeneity was anticipated and observed. Between-study variance τ^2^ was estimated using the restricted maximum-likelihood estimator (30), and Knapp–Hartung (31) adjustment was applied to calculate confidence intervals around pooled effects. Heterogeneity was quantified by the *I*^2^ index, with values of 25%, 50%, and 75% interpreted as low, moderate, or high inconsistency (32). Publication bias was explored by visual inspection of funnel plots, and asymmetries were verified by Egger's regression test when *k* > 10 (33).

Subgroup and meta-regression analyses explored effect modifiers and mediators of the insulin resistance and BGM association. Examined variables included metabolic condition (fasting vs insulin stimulation; primary outcome), insulin and glucose levels (combined mean and mean differences of group averages; secondary outcomes), study population (type 2 diabetes/prediabetes vs overweight/obese vs other insulin resistance-related conditions), BGM methodology (quantitative vs semiquantitative), BMI (combined mean of group averages), sex (% female), and age (combined mean of group averages).

Sensitivity analyses with the leave-one-out method were performed to estimate the influence of individual studies. Results were considered statistically significant when *P* < .05.

### Protocol Deviations

As very few of the included studies were longitudinal or intervention trials, we decided to use the Joanna Briggs Institute instrument for quality assessment instead of Grading of Recommendations, Assessment, Development, and Evaluations and National Institutes of Health tool for observational cohort and cross-sectional studies as predefined in our protocol. We restricted the meta-analysis to cross-sectional associations, thereby excluding within-group comparisons. We chose post hoc to group studies investigating individuals with type 2 diabetes and prediabetes, as they were often pooled in study populations. Subgroup analyses for cognitive status and ethnicity were omitted due to sparse reporting in the included studies.

## Results

### Study Identification and Selection

Titles and abstracts of 656 unique records from the literature search were screened. Of these, 78 reports underwent full-text screening; 51 met the predefined criteria. Fourteen reports were excluded due to overlapping study populations, 5 for unreported or unobtainable associations, and 1 for applying an irrelevant test condition. This left 31 reports for the systematic review. Of these, 7 reports were not included in the meta-analysis [Supplementary Fig. S2 and Table S2 (24)].

### Study Characteristics

The characteristics of the included studies in the systematic review are outlined in Table 1. Studies were published between 1990 and 2022 and included 4313 participants [1754 females (sex not reported in 5 studies)]. Participants numbers ranged from 11 to 1,666, mean age from 24 to 80 years, and mean BMI from 22 to 51 kg/m^2^. Studies were mainly performed in fasting state (71%), and insulin resistance was defined by either objective measures (29%) or clinical proxies (71%) of insulin resistance. Thirteen studies included participants with type 2 diabetes/prediabetes, 7 studies overweight/obese, and 11 studies were grouped as “other insulin resistance-related conditions” because of a mixture of participants ranging from healthy individuals or individuals with probable Alzheimer's disease, metabolic syndrome, the p.Pro50ThrAKT2 gene variant, polycystic ovary syndrome, or homeostatic model assessment for insulin resistance (>2.77) detected insulin resistance. In all cases, comparator groups had measurable or presumed lower levels of insulin resistance, such as metabolically healthy individuals. Quantification methods differed considerably among the 3 grouped study populations (percent quantitative studies, 38% vs 100% vs 45%). Four studies examined participants during more than 1 condition: 1 a somatostatin clamp with or without concurrent infusion of insulin at low dose and 3 with both fasting/saline-infusion and the hyperinsulinemic clamp. Two studies stratified participants by age or cognitive status, resulting in 29 observations (*k*) among the 24 studies in the meta-analysis.

**Table 1.** Characteristics of the 31 studies included in the systematic review grouped by study population

First author, year (study no.)	Study population	Metabolic condition	n	Age, years	Sex, %-female	BMI	Fasting glucose, mmol/L	IR measure	BGM methodology	Brain region(s)	Association
	Type 2 diabetes and Prediabetes										
Baker, 2011 (1)	PreD and T2D	Fasting	23	74 ± 7	–	27 ± 3	6.0 ± 0.7	HOMA-IR –	FDG-uptake (SUVR) *Ref.: Global level*	R-FC mCC	↓ ↓
Büsing, 2013 (2)	T2D No-T2D	Fasting	29 61	62 ± 14	–	–	9.1 ± 1.8 6.1 ± 2.5	–	FDG-uptake (SUVmax)	Not specified	↓
Eastman, 1990*^a^* (3)	T2D (Pima Indians) No-T2D (Pima Indians and Caucasian)	HIC and saline-Sham Glc: 5.6mM –	4 10	Pima Indians: 31-45yr Caucasian: mean 42yr	0 30	–	Pima Indians: – Caucasian: mean(range) 5.4(5.1-6.0)	–	CMRglc, (Gjedde-Patlak) LC: 0.418 IF: arterial	Not specified	T2D vs No-T2D: NS HIC vs Sham: NS
Eriksson, 2021 (4)	T2D PreD CON§	HIC Glc: 5.6mM Ins: 56mU/m^2^BSA/min	13 16 12	63[59;66] 66 [61;68] 61[56;64]	46 56 50	30[28;34] 31[28;33] 29[27;34]	8.0[7.5;10.3] 6.0[5.7;6.2] 5.5[5.3;5.6]	M-value 14.8[12.0;27.8] 29.4[18.5;31.8] 35.3[29.9;43.8] (μmol/kg/min)	CMRglc, (Gjedde-Patlak) LC: 0.65 IF: aorta	Not specified	T2D vs CON: ↑ PreD vs CON: NS
Garcìa-Casares, 2014 (5)	T2D	Fasting	25	60 ± 5	32	29 ± 4	–	HOMA-IR 3.6 ± 2.9	FDG-uptake (SUVR) *Ref.: Global level*	L-mTG (BA21) L-INS (BA13)	↓ ↓
Hirvonen, 2011 (6)	IGT CON	Fasting and HIC – Ins: 1mU/kg/min	13 9	49 ± 8 38 ± 12	62 44	38 ± 8 24 ± 2	5.9 ± 0.5 5.3 ± 0.5	M-value 11.9 ± 4.2 30.3 ± 6.0 (μmol/kg/min)	CMRglc (Gjedde-Patlak) – IF: arterial	WB, FC, lateralTC, mesialTC, PC, OC, INS, TH, ST, CBL	In IGT group BGM was HIC > fast (All regions except CBL), no change in hCON
Honkala, 2018*^b^* (7)	PreD and T2D	HIC – Ins: 40mU/m^2^BSA/min	21	49 ± 7	11	30 ± 4	6.8 ± 1.1	M-value 18.6 ± 12.3 (mmol/mL/kg)	CMRglc, (FUR) LC: 0.65 IF: arterialized	WB	↑↑
Ishibashi, 2015*^c^* (8)	IFG CON	Fasting	20 31	72 ± 5 68 ± 6	–	–	5.8 ± 0.1 4.9 ± 0.3	–	FDG-uptake (SUVR) *Ref.: VC/pCC*	PCu	↓
Képes, 2021*^d^* (9)	T2D Obese	–	51 45	51[12] 52[15]	–	33[8] 36[7]	7.2[2.6] 6.0[0.8]	–	FDG-uptake (SUVR) *Ref.: Global level*	PCu R-sFG	↓ ↓
Li, 2016*^e^* (10)	CH T2D No-T2D MCI T2D No-T2D AD T2D No-T2D	–	31 398 99 935 14 189	75 ± 1 74 ± 0 72 ± 1 73 ± 0 78 ± 2 75 ± 1	29 52 31 43 36 48	–	–	–	FDG-uptake (SUVR) *Ref.: Pons*	WB, FL, SMC, ST	CH NS MCI ↓ AD NS (All reported regions)
Li, 2022 (11)	40-60yr T2D CON‡ 60-80yr T2D CON‡	Fasting	34 34 37 32	51 ± 5 50 ± 5 66 ± 4 68 ± 5	9 21 27 28	25 ± 4 24 ± 4 25 ± 3 24 ± 3	8.4 ± 1.9 5.3 ± 0.6 9.2 ± 2.9 5.4 ± 0.6	–	FDG-uptake (SUVR) *Ref.: WB*	left triangle of iFG (region with significant agexT2D interaction)	40-60yr NS 60-80yr ↓↓
Roberts, 2014 (12)	T2D No-T2D	Fasting	154 595	80[76;84] 79[75;84]	38 45	–	6.4[5.6;7.3] 5.4[5.0;5.8]	–	FDG uptake, (SUVR) *Ref.: Pons*	WB pCC TG AG	↓↓ ↓↓ (↓) ↓↓
Waqas, 2019*^f^* (13)	T2D No-T2D*	Fasting	33 86	67 ± 4 66 ± 3	58 49	31 ± 6 26 ± 5	7.3 ± 1.3 5.5 ± 0.6	–	Corrected FDG-uptake (SUVglc)	WB	NS
	Overweight and obese										
Almby, 2021 (14)	RYGB pre vs post surgery	HIC Glc: 5.0mM Ins: 80mU/m^2^BSA/min	11	35 ± 8	64	40 ± 4 30 ± 4	6.0 ± 0.5 5.3 ± 0.5	M-value 7.6 ± 3.9 8.6 ± 2.5 (mg/kgLBM/min)	CMRglc (2-CM) LC. 0.65 IF: aorta/arterialized	WB	↑
Nummenmaa 2012*^g^* (15)	Morbidly obese NW†	HIC – Ins: 1mU/kg/min	19 16	46 ± 10 48 ± 10	–	44 ± 4 24 ± 2	–	–	CMRglc (Gjedde-Patlak) LC: 0.8 IF: arterialized	R-CN	↑
Orava, 2014 (16)	OW/obese NW	Fasting (warm condition)	17 24	40 ± 9 39 ± 10	71 79	33 ± 6 22 ± 2	–	–	CMRglc (Gjedde-Patlak) LC: 0.52 IF: aorta	CBL, FL, cortex, HYP, LL, OL, PL, Pons, sublobar regions, TL	NS
Tuulari, 2013 (17)	Morbidly obese (incl. T2D and PreD, *n* = 8) CON	Fasting and HIC - Ins: 1mU/kg/min	22 7	46 ± 9 48 ± 6	91 71	44 ± 3 24 ± 2	5.0 ± 0.3 4.9 ± 0.6	M-value 12.6 ± 5.8 37.2 ± 7.6 (–)	CMRglc (Gjedde-Patlak) LC: 0.8 IF: arterial	aCBL pCBL FL, LL, MB, OL, PL, TL	In obese group BGM was HIC > fast (All regions), no change in hCON
Wang, 2001 (18)	Morbidly obese NW/OW	Fasting	10 10	39 ± 7 38 ± 6	50 30	51 ± 5 25 ± 3	–	–	CMRglc (Gjedde-Patlak) – IF: arterial	WB, L-FC, L-PC, L-TC, L-OC, L-BG, TH, CBL	NS (All regions)
Wang, 2002 (19)	Morbidly obese NW*	Fasting	10 20	36 ± 10 35 ± 10	60 30	51 ± 4 21 ± 2	–	–	CMRglc, (Sokoloff model) LC: 0.52 IF: arterial	WB	(↑)
Wang, 2020 (20)	Obese NW/OW*	Fasting (No-stimulation)	16 11	32 ± 9 31 ± 6	0 0	39 ± 7 24 ± 3	–	–	CMRglc, (Sokoloff model) – IF: arterialized	L-MFG and L-sFG	↓
									Other insulin resistance-related conditions		
Anthony, 2006 (21)	IR (HOMA-IR ≥2.77) IS (HOMA-IR <1.55)	Somatostatin clamp +/− low-dose insulin at Fastinging levels	7 7	49 ± 10	0 0	28(24-35) 27(24-30)	5.7(5.1-6.6) 4.8(4.2-5.8)	HOMA-IR 6.3(2.9-9.5) 1.3(0.9-1.6)	CMRglc, (Gjedde-Patlak) – IF: arterial	WB, L-ventral striate, CBL, L-Amyg, PC	In both groups BGM was larger under insulin stim. (WB, L-ventral striate, PC; only IS: CBL, L-Amyg). Increase was greater in IS group (WB, PC).
Castellano, 2015 (22)	PCOS CON‡	Fasting	7 11	25 ± 4 24 ± 3	100	25 ± 2 24 ± 3	4.5 ± 0.3 4.1 ± 0.4	HOMA2-IR 0.7 ± 0.3 0.5 ± 0.3	CMRglc (Gjedde-Patlak) – –	GM, sFC, mFC, Orbito-FC, sPC, iPC, SMG, sTC, mTC, iTC, EC, ParaHIP, TH, Caudate, HIP, Amyg	↓ (mFC, SMG, mTC, other regions NS)
Chen, 2022 (23)	CH	Fasting	189	49 ± 9	32	25 ± 3	5.8 ± 1.2	HOMA-IR 2.0 ± 1.6	FDG-uptake (SUVR) *Ref.: Left cuneiform leaf*	WB	↓
Ennis, 2021 (24)	Healthy	Fasting	69	64 ± 5	58	–	5.2 ± 0.3	HOMA-IR 0.9 ± 0.3	FDG-uptake (SUVR) *Ref. cerebellar GM*	FC, lateralTC, SMG, AG, pCC	NS (All regions)
Femminella, 2021 (25)	Probable AD	Fasting	130	72 ± 7	39	26 ± 4	4.9 ± 0.5	HOMA2-IR 1.1 ± 0.9	CMRglc (Spectral analysis) LC: 0.48 IF: arterial	HIP	↓
Ishibashi, 2017 (26) (34)	Healthy, NW	Fasting	59	76 ± 6	83	–	5.7 ± 0.5	HOMA-IR 1.0 ± 0.5	FDG-uptake (SUVR) Ref. cerebral cortex	PCu	NS
Kantonen 2022*^h^* (27)	High obesity risk Low obesity risk	HIC – Ins: 40mU/m^2^BSA/min	19 22	27 ± 4 23 ± 3	0	27 ± 2 22 ± 2	5.5 ± 0.4 4.9 ± 0.5	HOMA-IR 2.2 ± 0.8 1.2 ± 0.7	CMRglc (FUR) LC: 0.65 IF: arterialized	Not specified	↑ (Frontotemporal and cingulate cortices, HYP, and bilaterally in insula and putamen)
Latva-Rasku 2018 (28)	p.Pro50Thr AKT2-carriers Noncarriers‡	HIC – Ins: 40mU/m^2^BSA/min	20 25	62 ± 6 64 ± 5	0 0	29 ± 3 28 ± 3	6.1 ± 0.3 6.0 ± 6.5	M-value 17.6 ± 10.3 29.2 ± 15.2 (μmol/kg/min)	CMRglc (FUR) LC: 0.65 IF: arterialized	FL, TL, limbic system, PL, OL, MB, CBL	↑↑ (all regions)
Nam, 2017 (29)	MetS No-MetS	Fasting	50 214	Mean: 46 (38-65)	0 0	–	–	–	FDG-uptake (SUVmax)	FC (ROI: 2 × 5 cm)	↓↓
Osborne, 2019 (30)	High BGM Low BGM (in Amyg)	Fasting	232	55[44-64]	58	26[23;31]	5.3[4.9;5.8]	–	FDG-uptake (SUVR) *Ref. mean TL*	Amyg	High amygdala BGM at baseline predicted new onset diabetes
Willette, 2015 (31)	CH middle-aged (incl. T2D, *n* = 7)	Fasting	150	61 ± 6	72	28 ± 5	5.3 ± 0.6	HOMA-IR 2.2 ± 1.9	FDG-uptake (SUVR) *Ref. R-cuneus*	WB L- MTL	↓ ↓

Data depicted as mean ± SD, median (range), or median [interquartile range]. The arrows under the association column, indicate if more pronounced insulin resistance (determined either by insulin resistance measure or by study population eg, type 2 diabetes vs control) is associated with higher (↑) or lower (↓) brain glucose metabolism, and the strength of association was illustrated by (↑)/(↓) for trend *P* = .05, and one or two arrows for *P-*values <.05 and <.01, respectively.

Abbreviations: a, anterior; AD, Alzheimer's disease; AG, angular gyrus; Amyg, amygdala; BA, Brodmann area; BG, basal ganglia; BGM, brain glucose metabolism; BMI, body mass index; BSA, body surface area; CBL, cerebellum; CC, cingulate cortex; CH, cognitively healthy; CMRglc, cerebral metabolic rate of glucose; CN, caudate nucleus; CON, control subjects; EC, entorhinal cortex; FC, frontal cortex; FG, frontal gyrus; FL, frontal lobe; FUR, fractional uptake rate; glc: target glucose; GM, grey matter; HIC, hyperinsulinemic-euglycemic clamp; HIP, hippocampus; HOMA-IR, homeostatic model assessment for insulin resistance; HYP, hypothalamus; i, inferior; IF, input function; IFG, impaired fasting glucose; IGT, impaired glucose tolerance; Ins, insulin infusion rate; INS, insula; IR, insulin resistant; IS, insulin sensitive; L-, left; LC, lumped constant; LL, limbic lobe; m, middle; M, medial; MB, midbrain; MCI, mild cognitive impairment; MetS, metabolic syndrome; NS, not significant; NW, normal weight; OC, occipital cortex; OL, occipital lobe; OW, overweight; p, posterior; PC, parietal cortex; PCOS, polycystic ovary syndrome; PCu, precuneus; PL, parietal lobe; PreD, prediabetes; R-, right; ROI, region of interest; RYGB, Roux-en-Y gastric bypass; s, superior; SMC, sensory motor cortex; SMG, supramarginal gyrus; ST, striatum; SUV(R), standardized uptake value (ratio); T2D, type 2 diabetes; TC, temporal cortex; TG, temporal gyrus; TL, temporal lobe; TH, thalamus; UNS, unspecified; VC, visual cortex; WB, whole brain; Yr, years.

^*a*^Two Pima Indians without diabetes were not studied under HIC; the control group included 4 (all male) Pima Indians and 6 (3 males) Caucasians without diabetes.

^*b*^Values are combined average of intervention groups at baseline.

^*c*^IFG defined as fasting blood glucose between 100 and 110 mg/dL.

^*d*^Values reported as median [interquartile range], and pre-positron emission tomography glucose inserted as fasting glucose, though unknown if fasting.

^*e*^Values reported as mean ± SD but are most likely mean ± SEM.

^*f*^T2D is defined as fasting blood glucose <10 mM and non-T2D as fasting blood glucose <7 mM.

^*g*^Five out of the 19 subjects with T2D were excluded from ^18^F-2-fluoro-2-deoxy-D-glucose-positron emission tomography analysis due to treatment with glucose-lowering medication.

^*h*18^F-2-fluoro-2-deoxy-D-glucose-positron emission tomography ([^18^F]-FDG-PET) was only performed on 19 out of 22 in the low-risk group.

^*^Age-matched.

^†^Age and height matched.

^‡^Age and BMI matched.

^§^Age, sex, and BMI matched. References are listed in Supplementary Table S4 (24).

### Quality Assessment

Fifteen studies included in the meta-analysis were evaluated as low risk of bias and the remaining 9 studies as moderate risk [Supplementary Table S3 (24)]. The percentage of low-risk studies was 61% in the fasting state and 67% in the insulin-stimulated state.

### Insulin Resistance Exerts Distinct Effects on BGM During Fasting and Insulin Stimulation

**Figure 1.**
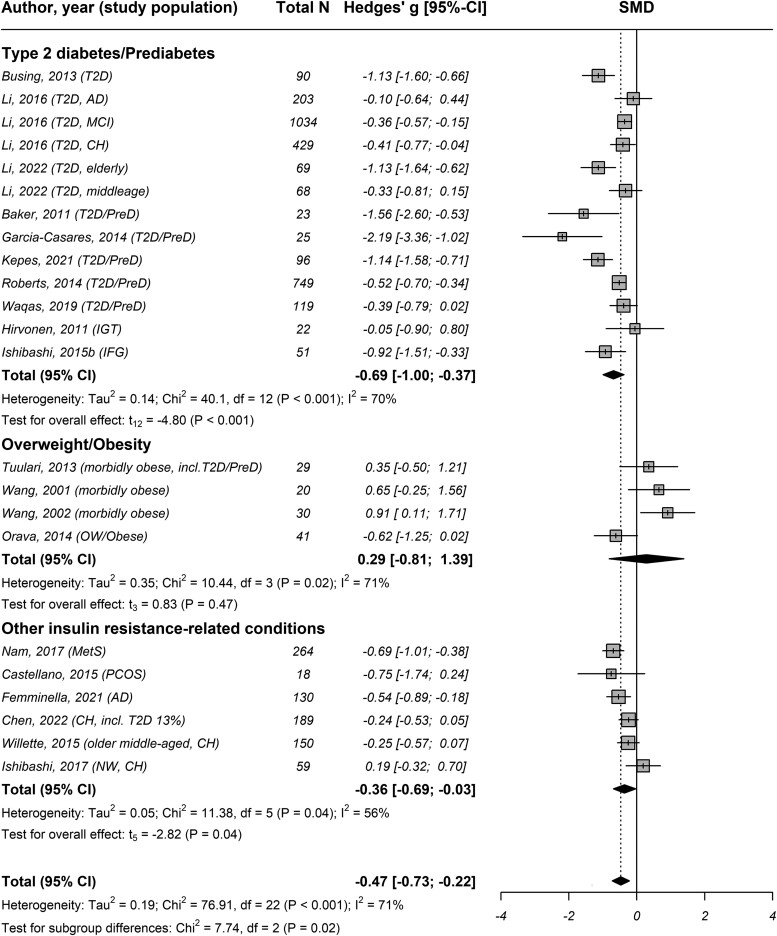
Forest plot of the association between insulin resistance and brain glucose metabolism of studies conducted during fasting stratified by study population. Abbreviations: AD, Alzheimer's disease; CH, cognitively healthy; IFG, impaired fasting glucose; IGT, impaired glucose tolerance; MCI, mild cognitive impairment; MetS, metabolic syndrome; N, number of study participants; NW, normal weight; OW, overweight; PCOS, polycystic ovary syndrome; preD, prediabetes; SMD, standardized mean difference; T2D, type 2 diabetes.

**Figure 2.**
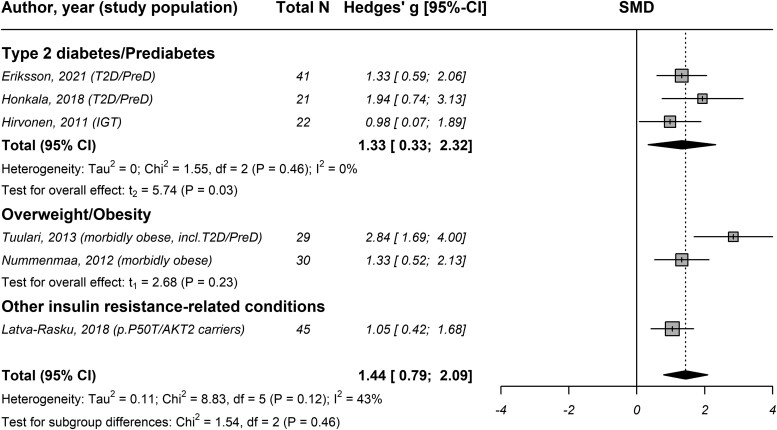
Forest plot of the association between insulin resistance and brain glucose metabolism of studies conducted during insulin stimulation from hyperinsulinemic clamps stratified by study population. Abbreviations: IGT, impaired glucose tolerance; N, number of study participants; preD, prediabetes; SMD, standardized mean difference; T2D, type 2 diabetes.

The pooled analysis of all 24 studies (*k* = 27) revealed high between-study heterogeneity [τ^2^ = 0.91 (95% confidence interval [CI] 0.56 to 2.15), *I*^2^ = 86% (95% CI 81 to 90), *P* < .001], with a significant difference between fasting vs insulin stimulation [χ^2^ = 47.39, *P* < .001; Fig. S3 (24)] Studies evaluating the effect of insulin resistance on BGM in a fasting state found BGM to be 0.47 SD lower on average [95% CI −0.73 to −0.22, *k* = 23, n = 3908, *P* < .001, *I*^2^ = 71% (95% CI 57 to 81); Fig. 1] in individuals with insulin resistance compared to control subjects. Contrary, studies performed in an insulin-stimulated state demonstrated that BGM on average increased by 1.44 SD [95% CI 0.79 to 2.09, *k* = 6, n = 188, *P* = .002, *I*^2^ = 43% (95% CI 0 to 78); Fig. 2] in individuals with insulin resistance compared to control subjects.

Stratification by study population of studies conducted in a fasting state revealed significant subgroup differences (χ^2^= 7.74, *P* = .02; Fig. 1). Studies of populations with type 2 diabetes/prediabetes and other insulin resistance-related conditions showed decreased BGM by −0.69 SD [95% CI −1.00 to −0.37, *k* = 13, n = 2978, *P* < .001, *I*^2^ = 70% (95% CI 47 to 83) and −0.36 SD (95% CI −0.69 to −0.03, *k* = 6, n = 810, *P* = .037, *I*^2^ = 56% (95% CI 0 to 82)], respectively, while studies including overweight/obese found no such association (0.29 SD 95% CI −0.81 to 1.39, *k* = 4, n = 120, *P* = .181, *I*^2^ = 71% (95%CI 18 to 90)] compared to control subjects.

Test for differences between study populations examined under conditions of insulin stimulation was not performed due to low number of studies (*k* < 3) within subgroups.

Inspection of funnel plots and Egger's tests did not suggest publication bias [all *P* > .05; Supplementary Fig. S4 (24)].

### Influence of Circulating Glucose and Insulin Levels on BGM

**Figure 3.**
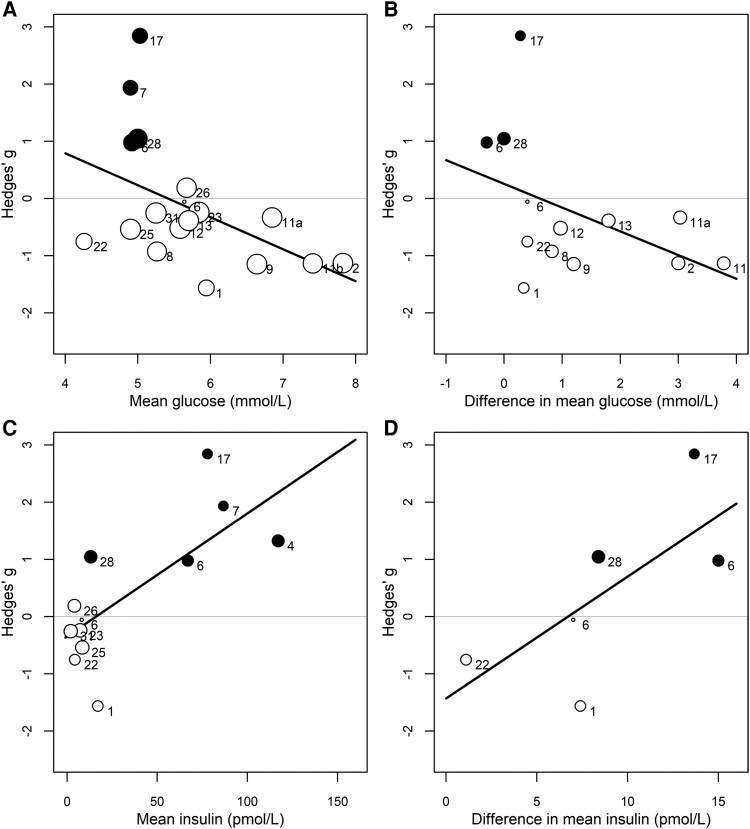
Bubble plots demonstrating the impact of (A) mean glucose (B) difference in mean glucose (C) mean insulin, and (D) difference in mean insulin on the association between insulin resistance and brain glucose metabolism. Plots include studies conducted during fasting (white circles) and insulin stimulation from hyperinsulinemic clamps (black circles). Numbers adjacent to circles indicate the study number referenced in Table 1.

Univariate meta-regression analysis revealed that studies with a higher mean glucose level had a stronger negative effect of insulin resistance on BGM [coefficient −0.56 (95% CI −1.10 to −0.02), *k* = 17, *P* = .042, *R^2^* = 28%; Fig. 3A], likewise, though insignificant, studies with a higher between group difference in mean glucose showed a stronger negative association between insulin resistance and BGM [coefficient −0.41 (95% CI −0.97 to 0.14), *k* = 12, *P* = .129, *R*^2^ = 19%; Fig. 3B]. The opposite was observed for mean and between-group difference of mean insulin, which resulted in stronger positive associations between insulin resistance and BGM [coefficients 0.02 (95% CI 0.01 to 0.04), *k* = 11, *P* = .010, *R*^2^ = 60%; Fig. 3C and 0.21 (95% CI −1.19 to 0.62), *k* = 5, *P* = .193, *R*^2^ = 35%; Fig. 3D, respectively].

### Meta-regression and Subgroup Analysis

In fasting conditions, subgroup analysis of BGM quantification method revealed that semiquantitative approaches overall reduced BGM in individuals with insulin resistance compared to controls [−0.56 SD (95% CI −0.82 to −0.30), *k* = 15], while quantitative measures showed no significant difference [−0.04 SD (95% CI −0.65 to 0.56), *k* = 7; Supplementary material Supplementary Fig. S5 (24)]. Univariate analysis, demonstrated a significant increase in the SMD with increasing mean BMI [coefficient 0.17 (95% CI 0.05 to 0.28), *k* = 19, *P* = .007, *R*^2^ = 36%], while mean age and %-female had no effect on the association between BGM and insulin resistance [coefficients −0.02 (95% CI −0.05 to 0.01), *k* = 27, *P* = .142 and 0.00 (95%CI −0.01 to 0.02), *k* = 24, *P* = .666, respectively].

### Sensitivity Analysis

Overall associations were unaffected by influential studies [Supplementary Fig. S6A-F (24)]. However, omitting Orava et al from the overweight/obese subgroup analysis of fasting studies showed a borderline positive association [0.65 SD (95% CI −0.06 to 1.37), *I^2^* = 0%]. Omitting any study from the insulin-stimulated subgroup analysis of type 2 diabetes/prediabetes and from the fasting subgroup analysis of other insulin resistance-related conditions, except Ishibashi et al 2017 (34), resulted in insignificant associations, likely due to the low number of included studies (*k* = 3 and *k* = 6, respectively).

Five fasting studies, which normalized FDG uptake to a global level, may have influenced our results as the global effects of insulin resistance on BGM may have been neutralized. However, excluding them only slightly reduced the SMD to −0.38 SD (95% CI −0.60 to −0.15, *I*^2^ = 61%).

## Discussion

This meta-analysis is the first to demonstrate the association between peripheral insulin resistance and BGM as highly dependent on metabolic conditions in which subjects are investigated. Studies with [^18^F]FDG PET scans performed during fasting demonstrate significant reductions in BGM in individuals with type 2 diabetes/prediabetes and other insulin resistance-related conditions, except obesity, when compared to metabolically healthy, normoglycemic, and/or lean individuals. Contrarily, studies conducted during hyperinsulinemic clamps show significant increases in BGM across all etiologies of insulin resistance. Our findings suggest circulating glucose and insulin as mediators of the observed variations, although there are caveats due to group effects and limited study numbers. Exploratory analyses revealed that the negative association between insulin resistance and BGM during fasting was only present for semiquantitative assessments. Furthermore, BMI, but not age and sex, affected the association between insulin resistance and BGM.

The observation that the metabolic condition (fasting vs insulin stimulation) was an important determinant of the association between insulin resistance and BGM was previously made by others (21), but few studies tested the hypothesis. Together, these studies demonstrated that insulin stimulation compared to the fasting state raises BGM in individuals with impaired glucose tolerance and severe obesity, with minor or no effect in lean normoglycemic individuals (35, 36). On the contrary, lowering insulin below fasting levels with somatostatin reduced BGM in metabolically healthy individuals (19, 20) but significantly less in insulin resistance (20). Altogether, these observations point to a vertical upward shift in the dose-response curve of insulin on BGM in individuals with insulin resistance (21). Our findings support these former observations but add the possibility of an additional rightwards shift of the dose-response curve in individuals with type 2 diabetes/prediabetes, compared to individuals with overweight/obesity.

Concerning brain insulin resistance, an increase in BGM in individuals with insulin resistance following insulin stimulation is counterintuitive and contrasts responses in skeletal muscle and adipose tissue, where insulin resistance is characterized by a notable reduction in insulin-stimulated glucose uptake (37). Though insulin sensitization would seem to be a straightforward explanation, it does not explain the lowering of BGM during fasting. Instead, some evidence suggests that delivery of insulin across the blood-brain barrier is disrupted with increasing insulin resistance (38). Insulin levels reached under a hyperinsulinemic clamp may thus be sufficiently high to overcome this disturbance. This being the case, we would expect that higher insulin levels were associated with increased differences in BGM. Our analysis was inconclusive, neither verifying nor falsifying the importance of peripheral insulin levels. Given the clear group effect, it is possible that actors other than insulin related to the hyperinsulinemic clamp may account for the paradoxical observation. A recent analysis of [^18^F]FDG PET scans performed under insulin stimulation also revealed no association between insulin and BGM (23). Adding to the complexity, administration of insulin to the brain by the intranasal route may elicit an increased response when measuring cerebral blood flow in insulin-resistant persons with obesity or type 2 diabetes (39, 40).

Glucose levels are also manipulated during hyperinsulinemic clamps and are often targeted at levels considered euglycemic in normoglycemic individuals, which in individuals with diabetes might resemble relative hypoglycemia. In hyperglycemic rodents, such normalization of blood glucose results in brain glucose hypometabolism (41), contrasting the increase in BGM observed in the 3 studies investigating individuals with dysglycemia under hyperinsulinemic clamps.

Our study found no reduction in BGM during fasting among individuals with obesity, unlike individuals with type 2 diabetes/prediabetes. Rather, 1 study reported elevated BGM in individuals with obesity, a finding corroborated by the similar trend identified in the sensitivity analysis. Additionally, increased BMI was associated with a greater positive difference in BGM. Likewise, previous studies found positive correlations between BMI and BGM (42, 43). Taking this into account, the findings suggest an inverted U-shaped relationship in BGM in the fasting state when progressing from metabolically healthy via obesity to overt type 2 diabetes. Equivalent, transient cerebral hypermetabolism in mild cognitive impairment preceding dementia have been reported (44, 45). Since only semiquantitative methodologies were associated with reduced BGM during fasting, the discrepancy between dysglycemia and obesity might be influenced by confounding from the applied quantification method. Notably, only 1 study employed quantitative methods in type 2 diabetes/prediabetes studies, while exclusively quantitative methods were used in studies of overweight/obesity. Therefore, both study population and quantification method could explain the differences.

This systematic review and meta-analysis has limitations. Despite the eligibility of 24 studies for the meta-analysis, we observed substantial heterogeneity among the studies, encompassing variations in examined populations, their characteristics, and the employed quantification methods for BGM. Stratification into subgroups was therefore deemed necessary, leading to few studies in each subgroup. Moreover, these subgroups were still mixed as some studies investigating the effects of obesity included subjects with dysglycemia and vice versa. Despite moderate-high heterogeneity, studies consistently aligned on 1 side of the reference line. Another limitation pertains to the uniform use of the hyperinsulinemic clamp for insulin stimulation. Consequently, the generalizability of the results to “normal” physiological insulin-stimulated conditions, such as post-meal states, may be limited. The cognitive status was not consistently determined across studies and might confound results given the potential influence of high risk of and existing cognitive diseases on BGM. We did not establish absolute effects of insulin resistance on BGM. As tomography settings, image processing, and quantification methods may impact BGM, we judged SMD as the best estimate despite its inherent limitations. Lastly, inclusion criteria were restricted to published reports, introducing the possibility of publication bias. Nevertheless, we adopted this approach to ensure the inclusion of reports subjected to rigorous peer review, thereby upholding the overall quality of the selected studies.

Conclusively, our findings suggest a link between insulin resistance and BGM that is significantly influenced by the metabolic condition of the subjects during [^18^F]FDG PET investigation. Notably, during insulin-stimulated conditions, all investigated types of insulin resistance showed enhanced BGM. Conversely, a fasting state reversed the relationship, except in obesity. The mechanisms of this paradoxical association remain uncertain including whether these responses are compensatory or maladaptive and should be investigated in future studies. This study further underlines the critical importance of carefully considering study designs, particularly regarding metabolic condition, the specific condition of insulin resistance under investigation, and the employed imaging technique, when conducting or interpreting future [^18^F]FDG PET studies.

## Data Availability

Some or all datasets generated during and/or analyzed during the current study are not publicly available but are available from the corresponding author on reasonable request.

## Prior Presentation

Parts of this study were presented in abstract form at the American Diabetes Association's 83rd Scientific Sessions, San Diego, CA, 2023.

## Study Registration

PROSPERO (CRD42022309231; https://www.crd.york.ac.uk/prospero/display_record.php?ID=CRD42022309231).
